# Tunable Faraday rotation of ferromagnet thin film in whole visible region coupled with aluminum plasmonic arrays

**DOI:** 10.1515/nanoph-2021-0327

**Published:** 2021-11-29

**Authors:** Feifei Zhang, Taisuke Atsumi, Xiaolun Xu, Shunsuke Murai, Katsuhisa Tanaka

**Affiliations:** Department of Material Chemistry, Graduate School of Engineering, Kyoto University, Katsura, Nishikyo-ku, Kyoto 615-8510, Japan; Light, Nanomaterials, Nanotechnologies (L2n), University of Technology of Troyes (UTT) & CNRS ERL 7004, 12 rue Marie Curie, Troyes, France

**Keywords:** aluminum plasmonic, blue, Faraday rotation (FR); magnetoplasmonics

## Abstract

To date, the plasmonic nanostructure utilized for magneto-optical (MO) enhancement has been limited to noble metals with resulted enhancement in the green-red part of visible spectrum. In this study, we fabricated a diffractive hexagonal array composed of Al nanoparticles (NPs) with a thin 7.5 nm ferromagnetic film and pushed the enhanced Faraday rotation (FR) into the blue to green range of the visible light. The freedom and ability to control the working spectral region in the whole visible range from 400 to 800 nm were also demonstrated by changing the lattice constant and the dielectric environment of plasmonic nanostructures. Particularly, in the blue range we obtained the maximum FR 0.57° at 410 nm with a broad boosting region around 0.5° from 400 to 500 nm. Moreover, the largest FR 1.66° was shown at 638 nm by tuning the dielectric environment into a higher refractive index medium. The results of our investigation demonstrate the potential of Al-based magnetoplasmonic effect and offer opportunities to push the MO spectral response out of visible range into the ultraviolet-blue range.

## 1 Introduction

**Figure 1:**
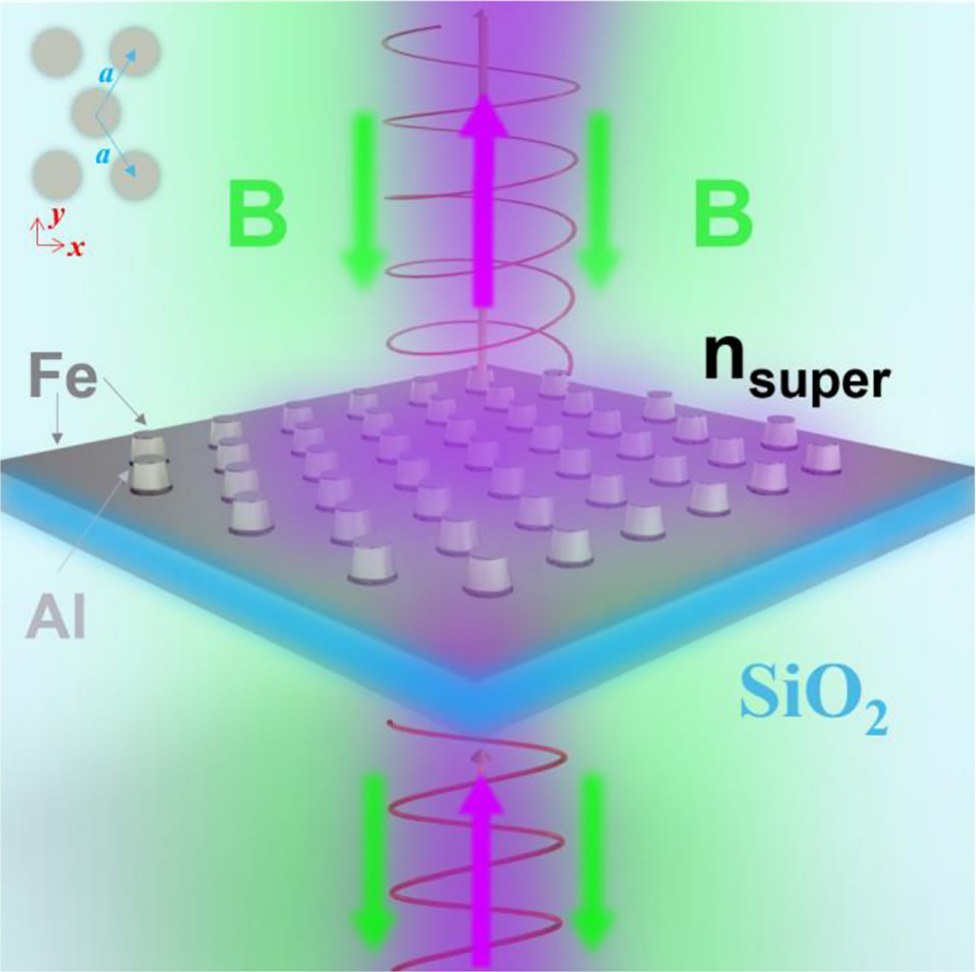
Schematic of Faraday rotation (FR) under the external magnetic field B along *z* direction on the Al NP hexagonal array with 7.5 nm Fe film on the top with different superstrates of refractive index *n*_super_. Left-top inset shows the unit cell of hexagonal array with pitch *a* in the *x-y* plane used in the calculation. The sketch of the Al nanostructure used in the FDTD calculations is provided in Appendix Figure S1.

As the main component of the modern optical telecommunication system, optical isolator/rotator has the ability to tune the polarization state of light, and the non-reciprocal behaviors are utilized to prevent the unwanted feedback in the laser cavity. The behind mechanism depends on the magneto-optical (MO) effects of magnetic materials such as Fe, Co, and Ni [1], [2], [3], [4]. Fundamentally, MO effects also provide physical information on the electronic and spin structures of materials [5, 6]. Depending on the transmitted or reflected configuration with different direction of magnetizations, various types of MO effects are distinguished like Faraday and Kerr effects. In this research, we will mainly concentrate on the Faraday rotation (FR) that refers the polarization rotation of transmitted light under the normal incident illumination with an external static magnetic field along the light wave vector, as shown by the schematic illustration in Figure 1.

Strong demands to enhance the MO effects exist for miniaturizing the MO devices like optical isolators and magnetic field sensors, however the typical FRs produced by the MO materials especially the traditional ferromagnetic metals are usually very small with high losses [2], [3], [4]. Metallic nanoparticles (NPs) have the ability to confine the light energy into its nanoscale vicinity with an enlarged intensity due to the collective oscillation of free electrons, therefore they show the appealing ability to enhance diversiform properties. In particular, many efforts have also been devoted to amplify the MO effect by plasmonic systems (so-called magnetoplasmonics) with the help of remarkably enhanced light–matter interaction. More advantages include the possibility to tune MO effects by exploring the sensitivity of plasmonic resonances to various factors like the geometry, arrangement, and dielectric environment, thus different plasmonic systems have been proposed to obtain giant MO effects. For instance, the combination of magnetic and plasmonic thin films that make use of propagating surface plasmon polaritons (SPPs) excited on the interface [7], [8], [9], [10], and plasmonic NPs that make use of localized surface plasmon resonances (LSPRs) [11], [12], [13], [14], [15] are two widely-adopted approaches. It has also been demonstrated that diffractive plasmonic nanostructures can largely enhance MO effects [16], [17], [18], [19], [20], [21], [22]. Such systems include periodic one-dimensional gratings and two-dimensional nanostructure arrays that support surface lattice resonances (SLRs), *i.e.*, hybridized modes between LSPRs and in-plane light diffraction [23], [24], [25]. When additionally combined with magnetic materials, these systems utilize two kinds of resonances for MO enhancement: the LSPRs that accumulate light field near the metallic surface enhance the MO effect locally, and the light diffraction that traps light inside the MO-active region further enhances the light–matter interaction. In this sense, diffractive plasmonic nanostructures possess the combined advantages of both diffraction-based, *i.e.,* magneto-photonic crystals and LSPR-based systems. In addition, these systems enjoy advanced properties including wavelength-selective MO enhancement by the periodicity and incident angles [26], [27], [28], [29], [30], [31], [32], [33], [34], [35], [36], [37]. In spite of a high degree of freedom in engineering the MO effect, the plasmonic material used in magnetoplasmonics has been thus far limited to Au and Ag [2], [3], [4]. Meanwhile, to date the FR enhancement in the experiment is typically obtained at the optical wavelengths of *λ* > 600 nm. Al has been proposed as a plasmonic material that can sustain LSPRs from the deep ultra-violet (UV) to the whole visible regions owing to its large carrier density [38], [39], [40], [41], [42]. Meanwhile, Al is especially advantageous for commercial applications because it is abundant in the Earth’s crust and compatible with the complementary metal–oxide–semiconductor (CMOS) process for the industrial production. Moreover, it is worthy to mention that dielectric nanostructures with high refractive index such as the representative material Si recently have also attracted enormous attention in the application of enhanced MO effects due to their reduced dissipative losses comparing to metallic materials [43], [44], [45], [46], [47]. The particular interest on the UV-blue MO effect is valuable in applications like the optical lithography system or other laser systems with short-wavelength light sources. Therefore, one major goal of this research is pushing the enhanced MO effects into UV-blue range. To date, most reported experimental results in magnetoplasmonics show a continuous MO enhancement within a 100 nm wavelength range due to the narrow Fano features utilized. And by exploring the lattice period and incident angles, the spectral tunability around 200 nm can be achieved [2], [3], [4]. Thus, we are also devoted to realize a broad spectral response and high tunability.

In this study, we fabricated Al NP hexagonal arrays to obtain the enhanced FR at shorter-wavelength spectral regions. The array was designed to show the hybridized lattice mode at around *λ* = 500 nm, overlapping with the LSPRs of Al NPs. As a proof of concept, Fe was chosen as the MO-active material in this research, considering its simple fabrication and larger saturation magnetic flux density [1]. A 7.5 nm Fe film was deposited on top of the fabricated Al NP array and the FR effect was examined. We further demonstrated the controllability of enhancement in the spectral region by the modulation of two types of modes, *i.e.*, LSPRs and SLRs resorting to changing the superstrate and lattice pitch. To confirm the experimental results, finite-difference time-domain (FDTD) method was also used to calculate the transmission spectra and FR spectra.

## 2 Methods

### 2.1 Fabrication of Al NP arrays

Hexagonal Al NP arrays with lattice pitch *a* = 400 and 460 nm (pitch *a* is illustrated by the inset of Figure 1) were fabricated using nanoimprint lithography in combination with reactive ion etching (RIE). The fabrication procedure is described as follows. Initially, a resist film was deposited on a 150 nm Al film on the quartz substrate. Next, the surface of the resist was nanostructured by nanoimprint techniques (Entre3, Obducat) to replicate the surface morphology of the Si mold, and the sample was then structured by RIE (Samco). After the nanoimprint processes, the uncovered part of the substrate was dry-etched under the flow of N_2_ and Cl_2_ gases (RIE-101iPH, Samco). The final size of the Al nanocylinder fabricated was examined by scanning electron microscopy (SEM, SU8000, Hitachi): height 150 nm and diameter 190 nm, respectively. Finally, a thin Fe film of 7.5 nm was deposited on the top of Al nanocylinder arrays by electron beam deposition (Eiko) under 4.0 × 10^−4^ Pa. The SEM images of Al NP array of *a* = 400 nm before and after the deposition of 7.5 nm Fe film are shown in Appendix Figure S2. The dielectric functions of Fe and Al film were determined by spectroscopic ellipsometry (FE-5000, Otsuka Electron) and the results are supplied as Appendix Figure S3. Moreover, the oxidation effect and long-term stability of Al NP was fully studied in our previous reports [37], [38], [39], [40]. It is better to mention that the height of Al NP was intentionally increased to enhance the forward scattering as reported by our previous research [40], and its crucial role in MO enhancement was also confirmed in different systems by another group [34]. Due to the high absorption of Fe, the thickness of Fe film was chosen to keep a good quality of film and with a relatively good transmittance.

### 2.2 Optical characterization

For the measurement of optical transmission as a function of incident angle *θ*_0_ (the angle in the free space, *i.e.,* the angle before light enters the quartz substrate), the sample was placed on a rotation stage, and white light from a light source (deuterium and halogen lamps, DH-2000, Ocean Optics) was made to be incident from the backside (quartz substrate side of refractive index *n*_quartz_) with polarization along *x* direction (as shown in Figure 1). The incident angle inside the glass substrate *θ*_in_ can be simply obtained by Snell’s law, *i.e.*, *θ*_in_ = arcsin(sin(*θ*_0_)/*n*_quartz_). The optical transmission spectra were obtained by normalizing with the transmission of the incident light through the area of pure quartz substrate, and plotted in the value from 0 to 1 in the following. To switch the superstrate environment from air (*n*_super_ = 1), two immersion oils (*n*_super_ = 1.46, 1.71) were used with another quartz coverslip.

The wavelength-dependence of FR angle (*θ*_F_) was measured at room temperature (∼20 °C) by the polarization modulation technique using a commercial system (K-250, Jasco) with a Xe lamp. A magnetic field of 1.5 T was applied perpendicular to the quartz substrate surface by an electromagnet as represented by Figure 1. The measurements were also carried out for quartz substrate in order to subtract the contribution of a diamagnetic substrate. A problem in evaluating the MO response of nanostructured solids such as magneto-photonic crystals is an optical anisotropy, *i.e.*, a structural birefringence that stems from their structures [18]. Anisotropy appears both intrinsically because of the symmetry in opal structures and extrinsically due to internal strains caused during the fabrication. This anisotropy causes optical birefringence and dichroism, which overlap the MO response in the measurement. In order to cancel those effects, we obtained the FR angle measured exactly at the same spot but applying an external magnetic field at two opposite directions, then the FR was obtained by *θ*_F_ = (*θ*_F_(1.5 *T*) − *θ*_F_(−1.5 *T*))/2.

### 2.3 FDTD calculation

**Figure S1:**
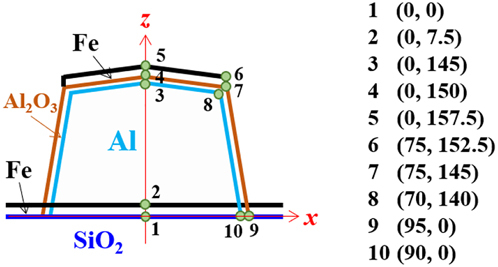
The sketch of Al nanostructure in the *x-z* plane (*y* = 0 nm) for finite-difference time-domain (FDTD) calculations, while the coordinates (*x*, *z*) of points from 1 to 10 are provided in the right side in unit of nm.

The optical scattering/transmission/absorption spectra and the FRs were calculated by the FDTD method (*Lumerical FDTD solutions*). The sketch of Al NP with a thin Fe film used in the FDTD calculation is illustrated in Appendix Figure S1. In detail, the Al NP was modeled with a 5 nm uniform oxide layer using a simplified model [39, 41], and the model is slightly tapered to take into account of the fabrication error. The forward scattering spectra for single Al NP and transmission/reflection spectra for periodic arrays were recorded with the light impinging from the substrate, then the absorption spectra were calculated by 1-transmission-reflection. For single Al NP, perfectly matched layers (PMLs) along the *x*-, *y*- and *z*-directions were employed with total-field scattering field source (polarization along *x* direction as shown in Figure 1). For the transmission spectra as a function of incident angle *θ*_in_, Bloch boundary conditions along the *x*- and *y*-directions, and PMLs along the *z*-direction were employed with *x*-polarized plane wave source (Broadband Fixed Angle Source Technique [BFAST]). The charge distribution and nearfield electric fields (|*E*|^2^) were recorded in *x*-*z* plane (*y* = 0 nm). In the FDTD calculations for the scattering and transmission/absorption spectra, experimentally measured permittivity of Fe was used as shown in Appendix Figure S3a. In all the scattering/transmission/absorption and MO calculations, the dispersive permittivity of Al determined from the ellipsometry (Appendix Figure S3b), and the ones of quartz and Al_2_O_3_ from the database of *Lumerical* software were considered [39], [40], [41], [42].

To calculate the FR angle by FDTD method, the permittivity tensor of Fe derived from Drude–Lorentz model equation was used and the corresponding derivation process are described as follows. The electron displacement vector $\vec{u}$ under the external static magnetic field $\vec{B}$ at time *t* is given by

(1) m d2u→dt2+mγ du→dt+mω02u→=q(E→+du→dt×B→)

where *m* is the electron effective mass, *q* = −1.6 × 10^−19^ C is the electron charge, *γ* is the probability of electron collision, and *ω*_0_ is the natural resonant frequency. Note that we actually assume that the material is isotropic here, therefore *γ* and *ω*_0_ are constant for all directions. If we apply the external static magnetic field along *z* direction $\vec{B}=\left(0,0,B\right)$ and take account of a harmonic time-dependence electric driving field $\vec{E}={\vec{E}}_{0}e^{-i\omega t}$ , a particular solution to describe the oscillation of the electron is $\vec{u}={\vec{u}}_{0}e^{-i\omega t}$ and ${\vec{u}}_{0}=\left(x_{0},y_{0},z_{0}\right)$ . Then, we obtain

(2) $$\left\{ \begin{array}{ccc} m\left(\omega^{2}+i\omega \gamma -\omega_{0}^{2}\right)x_{0}-i\omega qBy_{0} & = & -qE_{x}\\ m\left(\omega^{2}+i\omega \gamma -\omega_{0}^{2}\right)x_{0}-i\omega qBy_{0} & = & -qE_{y}\\ m\left(\omega^{2}+i\omega \gamma -\omega_{0}^{2}\right)z_{0} & = & -qE_{z} \end{array}\right.$$

The displaced electrons contribute to the macroscopic polarization $\vec{P}=Nq\vec{u}$ , where *N* is the electron density. The electric displacement $\vec{D}$ is associated with $\vec{E}$ by the relative dielectric constant *ε* by

(3) $$\vec{D}=\epsilon \epsilon_{0}\vec{E}=\epsilon_{0}\vec{E}+\vec{P}$$

where *ε*_*0*_ = 8.854 × 10^−12^ Fm^−1^ is the electric permittivity of free space. Therefore, by combining Eqs. (2) and (3) we can obtain

(4) $$\epsilon =\left(\begin{array}{lll} \epsilon_{xx} & -\epsilon_{xy} & 0\\ \epsilon_{xy} & \epsilon_{yy} & 0\\ 0 & 0 & \epsilon_{zz} \end{array}\right)$$

(5) $$\left\{ \begin{array}{l} \epsilon_{xx}\left(\omega \right)=\epsilon_{yy}\left(\omega \right)=1-\omega_{p}^{2}\frac{\omega^{2}+i\omega \gamma -\omega_{0}^{2}}{{\left(\omega^{2}+i\omega \gamma -\omega_{0}^{2}\right)}^{2}-\omega^{2}\omega_{c}^{2}}\\ \epsilon_{xy}\left(\omega \right)=\omega_{p}^{2}\frac{i\omega \omega_{c}}{{\left(\omega^{2}+i\omega \gamma -\omega_{0}^{2}\right)}^{2}-\omega^{2}\omega_{c}^{2}}\\ \epsilon_{zz}\left(\omega \right)=1-\omega_{p}^{2}\frac{1}{\omega^{2}+i\omega \gamma -\omega_{0}^{2\;}} \end{array}\right.$$

where $\omega_{p}=\sqrt{\frac{Nq^{2}}{\epsilon_{0}m}}$ is the plasma frequency, $\omega_{c}=\frac{qB}{m}$ is the cyclotron angular frequency from the Lorentz force. To calculate the FR, *ω*_p_ of Fe as 10.07 eV (about 253 nm), *γ* = 1.6 eV, *m* = 1.2 × 9.11 × 10^−31^ kg were adopted [5]. *ω*_0_ = 1.5 eV, B = −300 T were used, and the huge difference on the magnetic field intensity with experimental value is because Eqs. (4) and (5) are derived for the paramagnetic materials not for the ferromagnetic materials [46]. The calculated diagonal and off-diagonal elements of the permittivity tensor of Fe used in MO calculations are shown in Appendix Figure S3c–e. In order to incorporate the permittivity tensor of Fe (Eqs. (4) and (5)) into the *Lumerical* software for MO calculations, the unitary transformation to make the permittivity tensor diagonal and combination with grid attribute objects were used. Furthermore, the sweep of dispersive permittivity tensor of Fe was used in a step of 2 nm from 350 to 800 nm to get the wavelength dependence of FR angle in the FDTD calculation. The FR angle *θ*_F_ in the MO calculation was calculated by $\theta_{\text{F}}=\text{arctan}\left(\tilde{E_{y}}/\tilde{E_{x}}\right)$ where $\tilde{E_{y}}$ and $\tilde{E_{x}}$ are averaged electric field component along the *y*- and *x*-direction respectively recorded by the transmission monitor.

## 3 Results and discussion

To fully explore the advantage of broadband scatter of Al NP, the geometry of Al NP was optimized aiming to display a broadband spectral tunability [39], [40], [41], [42]. To start with, the forward scattering spectra of single Al NP with Fe film are plotted as a function of *n*_super_ in Appendix Figure S4. When *n*_super_ = 1, only one broad peak arises around 454 nm in the spectral range from 350 to 800 nm. When switching *n*_super_ to 1.46 and 1.71, this peak redshifts to 592 and 636 nm respectively, accompanied with a higher mode at the shorter wavelength side below 450 nm. The color-coded forward scattering spectra demonstrate the tunability to switch the LSPR window by changing the dielectric environment around Al NP. By investigating the charge distribution of these two modes as shown in the right of Figure S4, the complex behaviors far more than dipole and quadrupole are revealed [41, 42]. This is due to the complicated structures that we are considering in the calculation: the Al_2_O_3_ layer and Fe film on the bottom and top of Al NP. The influence of this nanostructure on the optical properties will be discussed later.

**Figure 2:**
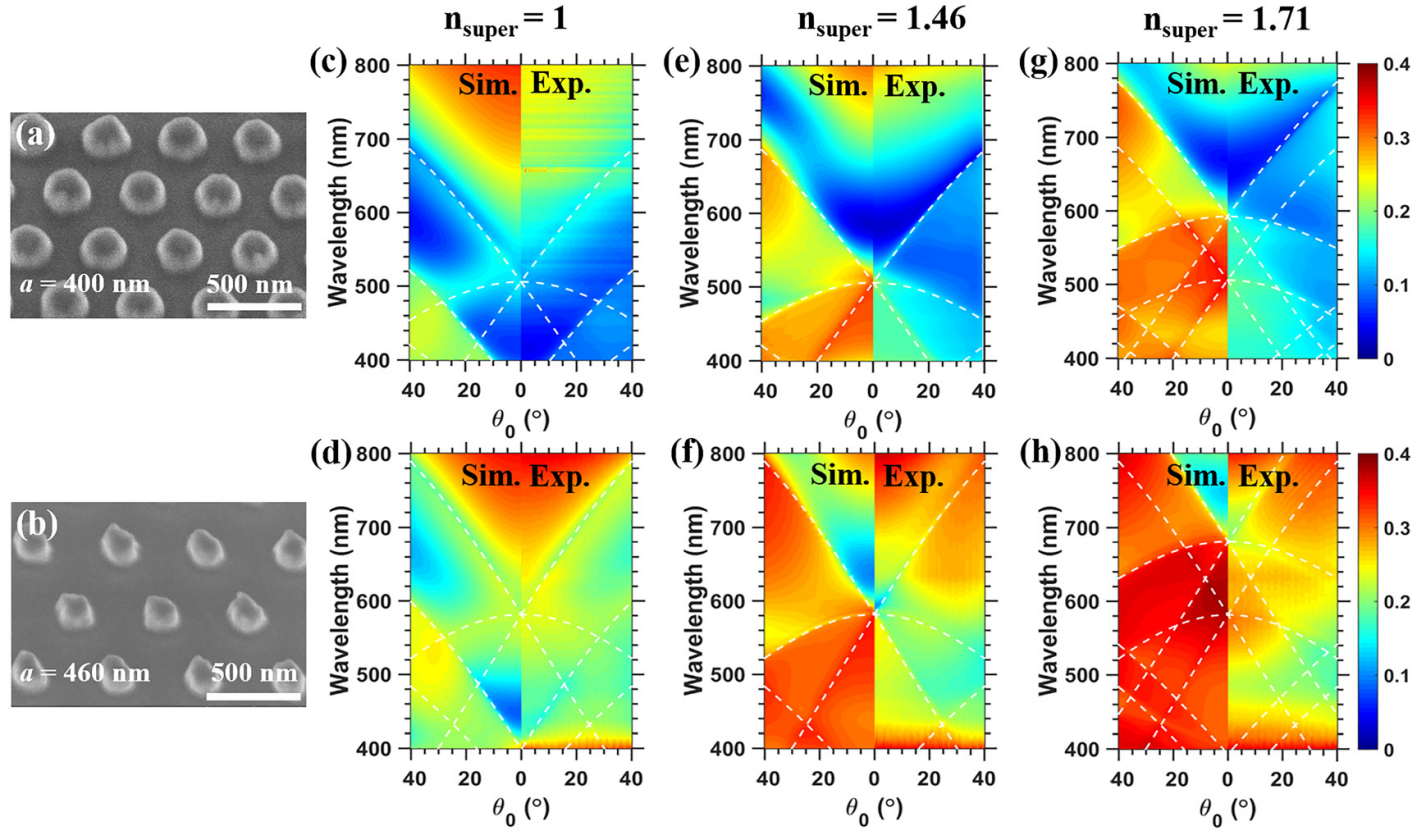
The 30°-tilted scanning electron microscopy (SEM) images of Al nanoparticle (NP) hexagonal array after the deposition of 7.5 nm Fe film with lattice constant *a* = 400 nm (a) and 460 nm (b). The calculated (Sim.) and experimental (Exp.) transmission spectra as a function of *θ*_0_ for the Al NP array with a 7.5 nm thick Fe film on the top with *n*_super_ = 1, 1.46, and 1.71 respectively for *a* = 400 nm (c, e, g) and 460 nm (d, f, h). White dashed lines indicate the conditions of in-plane diffraction (the specific modes are indicated in Appendix Figure S5), and the color bar is uniformly indicated on the right side of each row.

**Figure S2:**
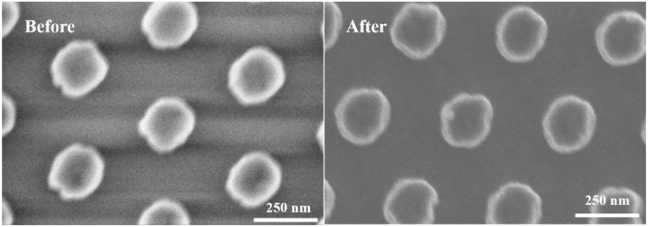
The scanning electron microscopy (SEM) image of Al nanoparticle (NP) array with *a* = 400 nm before (left) and after (right) deposition of 7.5 nm Fe film on the top.

Figure 2a and b show the SEM images of Al NP hexagonal arrays with lattice constant *a* = 400 and 460 nm after the deposition of 7.5 nm Fe film, while Appendix Figure S2 shows the SEM images of Al NP hexagonal arrays with *a* = 400 nm before and after the deposition of 7.5 nm thick Fe film. When arranged into periodic array, the lattice coupling with the LSPRs of single NP appears [23], [24], [25, 41, 42]. To verify the wavelength of lattice mode or SLR in the hexagonal array, the experimental zeroth-order transmittance measurements were done as a function of *θ*_0_, where *θ*_0_ was varied to give momentum in the *x* direction (see the inset of Figure 1 for *x* direction). Based on the conservation of the parallel component of the wave vector in the NP array plane, the Rayleigh anomaly conditions satisfy the relation: ***k***_out_ = ***k***_in_ ± ***G***, where ***k***_out_ and ***k***_in_ are the in-plane component of diffracted and incident wave vectors, respectively. ***G*** = (m_1_ ***b***_***1***_, m_2_ ***b***_***2***_) is the reciprocal lattice vector of the NP array, which can be expressed for the hexagonal lattice as [48]:

(6) $$\begin{array}{l} b_{1}=\left(2\pi /a\right)\left(x+y/\sqrt{3}\right)\\ b_{2}=\left(2\pi /a\right)\left(x-y/\sqrt{3}\right) \end{array}$$

where *m*_*1*_ and *m*_*2*_ are integers defining the diffraction order. When ***k***_in_ does not have a component in the *y*-direction, as is true in the present case, ***k***_out_ can be expressed as:

(7) $$k_{\text{out}}^{2}=k_{\text{in}}^{2}+2\left(2\pi /a\right)\left(m_{1}+m_{2}\right)k_{\text{in}}+{\left(2\pi /a\right)}^{2}{\left(m_{1}+m_{2}\right)}^{2}+{\left(2\pi /a\right)}^{2}{\left(m_{1}-m_{2}\right)}^{2}/3$$

where *k*_out_ = 2π*n*/*λ* and *k*_in_ = (2π*n*_quartz_/*λ*)sin*θ*_in_ are, respectively, the parallel components of the diffracted and incident wave vectors, *n* is the refractive index of different diffracted environments either to be *n*_quartz_ or *n*_super_, and *n*_quartz_ ≈ 1.46 is the refractive index of quartz. Note that we denote the magnitude of ***k*** by *k*. According to Eq. (7), the specific attribution to each lattice mode is provided in Appendix Figure S5. When the substrate and superstrate have a mismatch on the refractive index, a partial SLR through either the superstrate or the substrate exists, which are denoted as dashed and solid lines respectively in Figure S5 [25, 41].

In Figure 2, the experimental transmission spectra as a function of *θ*_*0*_ are displayed in row for varied lattice constant *a* and in column for varied *n*_super_, and the lattice mode wavelengths are also plotted in white dashed lines. When placed in a uniform dielectric environment, *i.e.,* with *n*_super_ = 1.46, the refractive index mismatch between the upper layer and the quartz substrate around the Al NP is reduced, facilitating the in-plane diffraction, therefore the least diffraction orders are shown in Figure 2e and f. More diffraction orders appear with varied *n*_super_ from 1.46 to 1 and 1.71 due to the partial SLRs through either the superstrate or the substrate, and the spectral shift is caused by the change of refractive index around the Al NP. According to Eq. (7), the specific attribution to each lattice mode is provided in Appendix Figure S4. As for *a* = 400 nm and *n*_super_ = 1 (Figure 2c), a prominent dip around *λ* = 410 nm at *θ*_0_ = 0° corresponds to the excitation of LSPR of the Al NPs. This main dip redshifts with increasing *θ*_0_, and the shifts follow the dashed lines (0, ±1) mode from the superstrate (Appendix Figure S5), representing the conditions for diffraction in the plane of the array. As for *a* = 460 nm and *n*_super_ = 1 (Figure 2d), the dip is redshifted to 430 nm due to the shifting of lattice coupling with increasing lattice constant *a*. However, it also follows the (0, ±1) lattice mode from the superstrate. Similarly, the angular profiles of the main dips follow well the different diffraction orders with varying *a* and *n*_super_ in Figure 2e–h, indicating the hybridization of LSPRs with in-plane diffraction, *i.e.*, SLRs. The symmetric behavior between calculated and experimental spectra as plotted in Figure 2c–h confirms the same phenomena, and proves a good accordance with each other. Furthermore, the transmission intensity shows a value below 0.4 even in the calculation, and the relatively higher transmission of array with *a* = 460 nm (Figure 2d, f, and h) than the one with *a* = 400 nm (Figure 2c, e, and g) is owing to the reduced NP number density [40]. More detailed analysis on the mode coupling for lattice modes in the Al NP array are available in the previous researches [23], [24], [25, 39], [40], [41], [42]. Complex features on the nature of plasmonic resonances may arise when we play with the polarization and *θ*_0_ on the hexagonal lattice as pointed out by other groups [36, 37]. With respect to our main purpose on FR, at the normal incidence our hexagonal nanostructure shows very little difference on the transmission spectra by using *x*- or *y*-polarized light.

**Figure S3:**
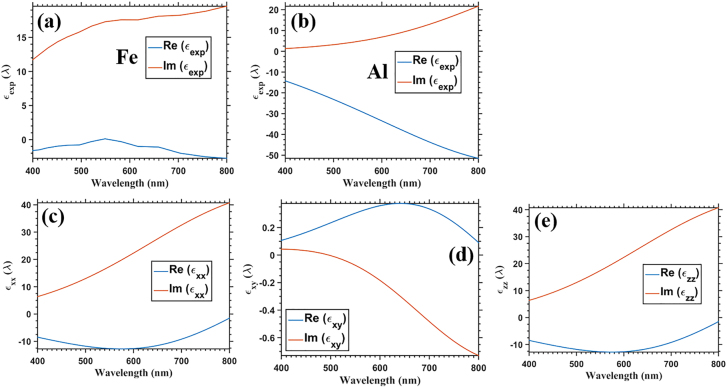
Dielectric functions of Fe (a) and Al (b) film experimentally determined by spectroscopic ellipsometry. (c–e) Permittivity tensor of Fe in the MO calculation from Eqs. (4) and (5). The real parts (Re) are in blue lines, while the imaginary parts (Im) are in brown lines.

Since main concerns may arise on the possibility of SPPs on the surface of Fe film, here we provide several reasons to simplify our situations without SPPs: Firstly, SPPs more likely happen on the metallic film with free electrons like Au, Ag, and Al. Fe is a ferromagnetic metal, thus it is usually not a good material for SPP [1]. If we check the measured dielectric function of Fe in Appendix Figure S3a, we can see the absolute value of its imaginary part is much larger than the one of its real part. Thus, according to the frequently adopted equation to calculate the SPP wavelength *λ*_spp_ = *λ*_0_ ((*ε* + *ε*_m_)/εε_m_)^1/2^, where ε_m_ is the real part of the dielectric function of Fe as displayed in Figure S3a and *ε* is the dielectric function of the dielectric medium, *i.e., ε* = (n_super_)^2^, we will most probably obtain an imaginary value of *λ*_spp_ in the range of 400–800 nm. Secondly, Fe film is only 7.5 nm here, considering that the height of Al nanostructure is 150 nm. Thus, the influence of SPP will be quite small. Thirdly, according to Eq. (7) the obtained in-plane diffraction wavelengths in Figure S5 already show a good coincidence with the Fano features in both the calculated and experimental transmission spectra as shown in Figure 2. Lastly, we calculate different nanostructure setups to peel off the influence of each part subsequently as shown in Appendix Figure S6. The Fe nanohole array (black box 4) only creates a very small kink in the transmission spectrum (black line spectrum in Figure S6a), which is better shown in the absorption spectrum in Figure S6b. Meanwhile, this kink is also well located at 505.8 nm as denoted by the vertical black dashed line, exactly predicted by Eq. (7) for the in-plane diffraction wavelength. Thus, we can safely exclude the influence of SPP in this research.

**Figure 3:**
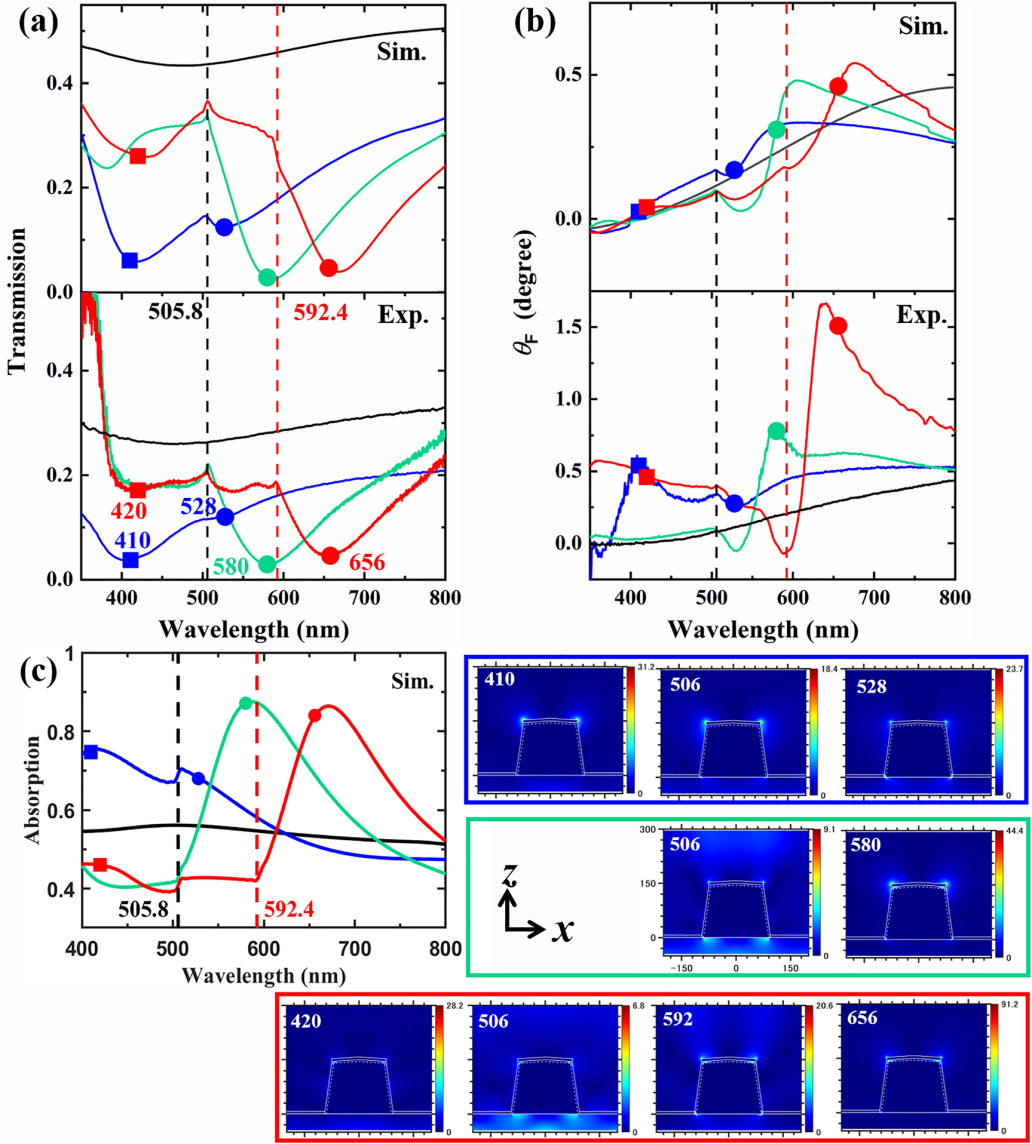
Calculated (upper panel, Sim.) and experimental (lower panel, Exp.) spectra of (a) optical transmission and (b) Faraday rotation (FR) angle *θ*_F_ of the Al nanoparticle (NP) array with *a* = 400 nm and 7.5 nm Fe film on the top at *θ*_0_ = 0° with different superstrates. Calculated absorption spectra are shown in (c), while the nearfield electric fields in *x*-*z* plane are plotted on the right side in colored boxes with the wavelengths noted on the top, and the colors of the boxes are in accordance with the colors of the line spectra. The direction and tick scale of *x-z* axis for the nearfield profiles are uniformly shown by one profile in the green box for the sake of the concision, and the white lines depict the nanostructure in conformity to Figure S1. The colors of spectra indicate *n*_super_ = 1 (blue), 1.46 (green), and 1.71 (red), while the black line spectra are the reference spectra of the Fe film with *n*_super_ = 1. The black vertical dashed line at *λ* = 505.8 nm denotes the condition of in-plane diffraction for all three different *n*_super_ (=1, 1.46, and 1.71), and the red vertical dashed line at *λ* = 592.4 nm denotes the in-plane diffraction for *n*_super_ = 1.71 for the red line spectra. The wavelengths of dips in the experimental transmission spectra (a, lower panel) are marked by square (at shorter wavelength) and circular marker (at longer wavelength) with their colors in accordance with the line colors of spectra. The corresponding wavelengths are indicated nearby the markers. To keep the uniformity, the same wavelength positions are also indicated by the same markers in the calculated transmission spectra (a, upper panel), FR spectra (b), and absorption spectra (c).

**Figure S4:**
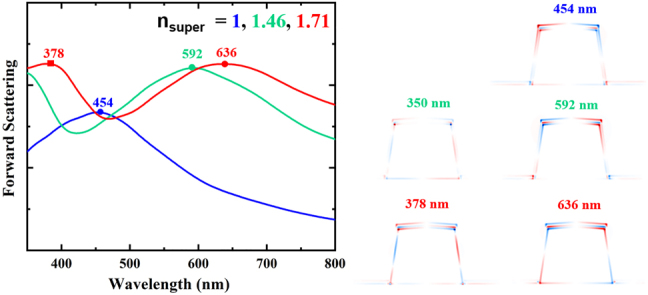
(Left) Forward scattering spectra of single Al nanoparticle (NP) with 7.5 nm Fe film. The line colors indicate the superstrate of *n*_super_ = 1 (blue), 1.46 (green), and 1.71 (red). (Right) Charge distribution at different peak wavelengths indicated by markers in the scattering spectra, and the wavelengths are noted nearby. The red and blue colors in the charge distribution mappings stand for the positive and negative charges respectively.

**Figure S5:**
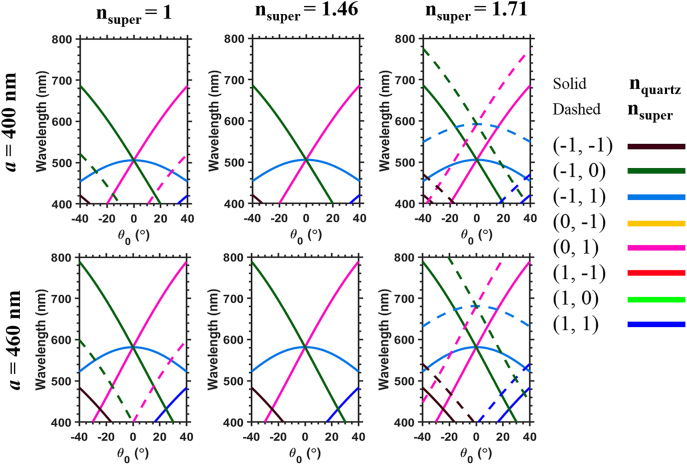
Lattice modes calculated from Eq. (7) with *n*_super_ = 1, 1.46, and 1.71 (in column) and *a* = 400 and 460 nm (in row) as a function of *θ*_0_. The lattice modes in the substrate environment (*n* = *n*_quartz_ in Eq. (7)) are denoted by solid lines, while the ones in the superstrate environment (*n* = *n*_super_ in Eq. (7)) are denoted by dashed lines. The corresponding orders are color-coded and plotted in the right. Note that the modes (0, −1), (1, −1), and (1, 0) overlap with modes (0, 1), (−1, 1), and (−1, 0), respectively.

The calculated and experimental transmission spectra for the array of *a* = 400 nm at the normal incidence (*θ*_0_ = 0°) with varying *n*_super_ are plotted in Figure 3a. For the Fe film (black solid line spectra in Figure 3a), only a shallow dip shows on the transmission spectra around 480 nm, while distinct dips appear with Al NP array (colored solid line spectra in Figure 3a) because of the strong LSPRs. For *n*_super_ = 1 (blue line spectra), beside a main dip around 410 nm in the experiment (blue square marker), another small dip at around *λ* = 528 nm appears (blue circular marker). This is because that the diffraction in the substrate side at 505.8 nm (vertical black dashed line) split the LSPR of single Al nanostructure about 454 nm (Figure S4) into two dips. The typical Fano lineshapes exist around 505.8 nm for all the three spectra due to the lattice mode coupling, while the main dips move from 410 to 580 (green circular marker) and 656 nm (red circular marker) when *n*_super_ increases from 1 to 1.46 and 1.71 owing to the redshifted LSPR of single Al nanostructure with increasing refractive index of background, as shown in Figure S4. As for *n*_super_ = 1.71, additional lattice mode (superimposed (0, 1), (−1, 0) and (−1, 1) modes from the superstrate side) appears at 592.4 nm, shown by the red vertical dashed line in Figure 3a. Both calculation and experiment confirm the distinct grating effect at the same wavelength, which is easily confirmed by the vertical dashed lines. In general, the calculated transmission spectra agree well with the experimental ones, although the LSPR positions marked by square and circular markers display a mismatch with each other. This difference can be attributed to the nanofabrication imperfections in the experiment, which cause internal defects inside the Al NPs and various disorder effects in the periodic array [39], [40], [41], [42]. Moreover, the highly damped higher mode below 500 nm in the case of *n*_super_ = 1.46 and 1.71 can be additionally ascribed to the surface roughness of NP [41]. It is worthy to note that the transmittance of 7.5 nm Fe film already shows a low value below 0.3 or 30% in the experiment, which can hinder the further application devoted to MO devices. Furthermore, the LSPRs and SPPs enhanced MO effects often accompany with a transmission damp, which has been recently reported to be improved resorting to the extraordinary transmission of nanoholes [35].

The comparison of calculated and experimental FR spectra for the Al NP array of *a* = 400 nm can be examined in Figure 3b. The reference of thin Fe film displays an almost linear increment of *θ*_F_ as a function of wavelength without any featured resonant behavior (increase from 0° at 400 nm to 0.44° at 800 nm in the experiment), as denoted by the black line spectra in Figure 3b. In contrast, *θ*_F_ of the Fe film on the Al NP arrays displays various resonant features. Therefore, we can readily trace the correlation between enhanced *θ*_F_ and the LSPRs or lattice modes due to the appearance of Al NP. The corresponding wavelengths of LSPRs and lattice mode from the experimental transmission spectra in Figure 3a are also indicated by the square/circular markers and vertical dashed lines respectively in Figure 3b. With the appearance of Al NP, the MO response shows a resonant modulation at the spectral vicinity of LSPRs where the minimum transmittance occurs approximately. In detail, the excitations of LSPRs of Al NPs produce the prominent peaks and dips in the experimental FR spectra: peak 0.57° at *λ* = 410 nm and dip 0.26° at *λ* = 534 nm for *n*_super_ = 1; dip −0.05° at *λ* = 529 nm and peak 0.81° at *λ* = 580 nm for *n*_super_ = 1.46; a boosting region around 0.5° from 400 to 500 nm due to the higher mode (red square marker), dip −0.07° at *λ* = 589 nm and peak 1.66° *λ* = 638 nm for *n*_super_ = 1.71. The spectral peak/dip shift of *θ*_F_ in Figure 3b comparing to the marker position in Figure 3a are mainly due to the varied dielectric function under the magnetic field, as shown by the variation on the dielectric function related to the external magnetic field (Eqs. (4) and (5)). Meanwhile, the obvious Fano lineshapes in the FR spectra around the markers’ wavelengths in the calculation and experiment are possibly associated with the quick phase transition across the LSPRs [34].

In the condition of in-plane diffraction denoted by the vertical lines at *λ* = 505.8 (black dashed vertical line) and 592.4 nm (red dashed vertical line), a modulation with Fano lineshape is found for the value of *θ*_F_. Bumps around *λ* = 505.8 nm of *θ*_F_ appears as 0.32° for *n*_super_ = 1, as 0.10° for *n*_super_ = 1.46, and as 0.39° for *n*_super_ = 1.71 in the experimental FR spectra. Meanwhile, the dip around *λ* = 592.4 nm in experimental *θ*_F_ for *n*_super_ = 1.71 can be also partially ascribed to the grating diffraction except for the nearby LSPR, while this wavelength is located between two small dips in the MO calculation (red line spectrum in the upper panel of Figure 3b). However, in the MO calculation distinct bumps are produced due to the SLRs at the wavelengths denoted by the vertical dashed lines in Figure 3b. The controllability of enhanced *θ*_F_ is demonstrated by shifting the enhanced region resorting to switching the immersion oil: with increasing *n*_super_ the enhanced region is redshifted. Particularly, in experiment we obtain enhanced *θ*_F_ to 0.57° at *λ* = 410 nm for *n*_super_ = 1, to 0.81° at *λ* = 580 nm for *n*_super_ = 1.46, to 1.66° at *λ* = 638 nm for *n*_super_ = 1.71, which indicates a stronger interaction of LSPRs during FR with the thin 7.5 nm Fe film. The UV and blue region have been rarely reported for enhanced MO effect, however with the Al NP array and the thin 7.5 nm Fe film we push the enhanced FR into the blue region. In spite of the different value of *θ*_F_, the calculated FR (Figure 3b, upper panel) shows similar features with experimental results. Both enhanced *θ*_F_ due to LSPRs and lattice modes are distinct, as indicated by spectral region close to the markers and vertical dashed lines. However, *θ*_F_ at the cutting-off wavelength of lattice mode (vertical dashed lines) seems to display weaker enhancement comparing to the spectral range of LSPRs (around square and circular markers). Beside the above-mentioned reasons for the mismatched transmission spectra between calculation and experiment, the mismatch in the FR spectra additionally comes from the deviations in the permittivity tensor between calculation and experiment, because it is significantly difficult to experimentally obtain an accurate estimation for the ferromagnetic material. Additional errors may come from the multiple reflections produced by the coverslip and immersion oil in the experiment to switch *n*_super_, which is impossible to consider in the FDTD calculations due to the unknown thickness of the oil film and the heavy need for computation memory.

**Figure S6:**
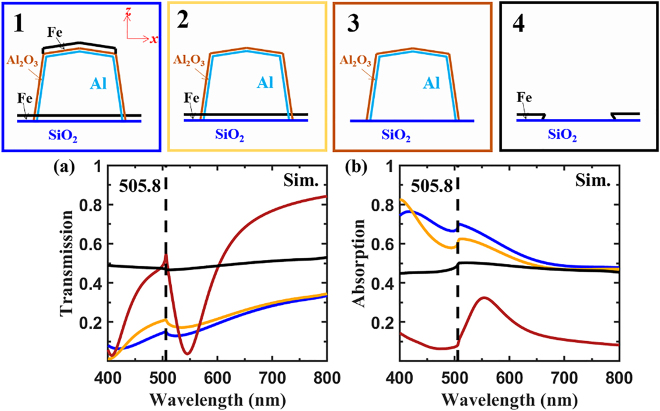
Calculated (a) transmission and (b) absorption spectra for the hexagonal array with *a* = 400 nm and *n*_super_ = 1 at normal incidence *θ*_0_ = 0° for different simulation setups of nanostructures as depicted above in colored and numbered boxes: 1. (blue box) The currently used setup to describe our experimental MO structure (7.5 nm Fe film was deposited after the Al nanoparticle (NP) array was fabricated); 2. (orange box) Based on structure 1, the Fe material on top of Al NP is removed; 3. (brown box) Based on structure 2, the Fe material on the quartz substrate is removed; 4. (black box) Based on structure 2, Al NP is removed, *i.e.*, Fe nanoholes are left. The vertical black dashed line is the in-plane diffraction wavelength as shown in Figure S5 for *a* = 400 nm and *n*_super_ = 1 according to Eq. (7).

To better understand the enhancement mechanism, the calculated absorption spectra are also plotted in Figure 3c. The calculated reference spectrum of Fe film only displays a slight bump around 500 nm. As the Al NP lattice appears, the kinks due to the occurrences of Rayleigh anomalies are distinctly visible as shown by the vertical lines, which is in the same manner as for the transmission curves in Figure 3a. As for the positions of markers in the calculated absorption spectra, they are well located in the vicinities of absorption peaks. The absorption is associated with the imaginary part of the dielectric function and electric field intensity [41], thus the strong absorption intensity usually means the strong localization of light field nearby the plasmonic nanostructures. This can be proved by the plotted electric field profiles on the right side that with a larger absorption value in the absorption spectra in Figure 3c a higher maximum value of the electric field intensity appears. The tiny hot spots near the Fe material on the top of Al NP corroborate the physical picture described above of the plasmon-assisted strengthened FR. We can further investigate the role of Fe material on the top of Al NP comparing to the Fe material on the substrate. Without the Fe material on the top of Al NP, the transmission dips (yellow line spectra in Figure S6a) become much shallower than the original one (blue line spectra in Figure S6a), and the absorption intensity decreases as shown in Figure S6b. Therefore, without the Fe material on the top of Al NP the plasmon enhanced MO effect is expected to be decreased because of the less localization of light energy. This can be also illuminated by the hot spots in the near field profiles in Figure 3c that strong hot spots are located on the top of Al NP, which is in strong contrast to the Fe material near the bottom of Al NP.

**Figure 4:**
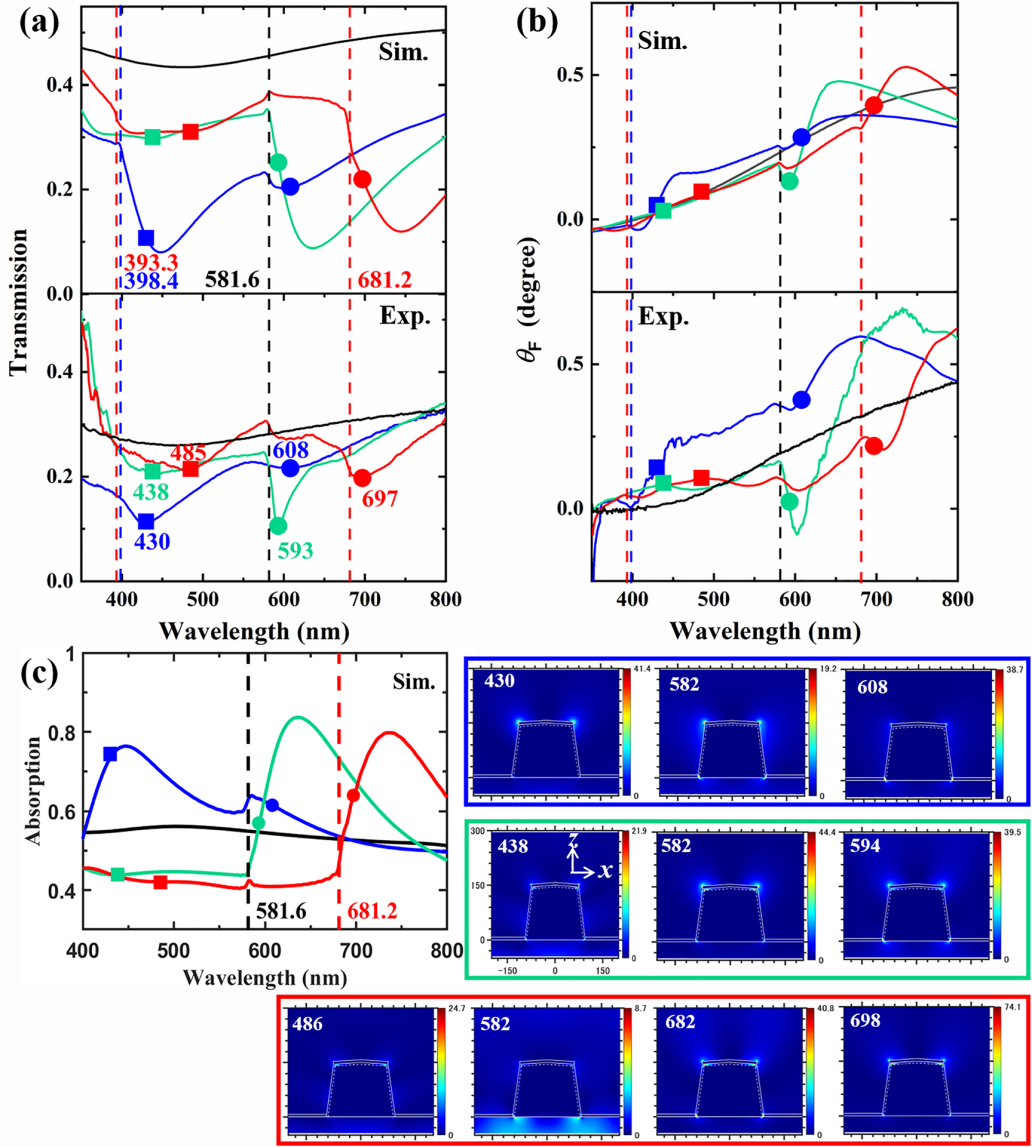
Calculated (upper panel, Sim.) and experimental (lower panel, Exp.) spectra of (a) optical transmission and (b) Faraday rotation (FR) angle *θ*_F_ of the Al nanoparticle (NP) array with *a* = 460 nm and 7.5 nm Fe film on the top at *θ*_*0*_ = 0° with different superstrates. Calculated absorption spectra are shown in (c), while the nearfield electric fields in *x*-*z* plane are plotted on the right side in colored boxes with the wavelengths noted on the top, and the colors of the boxes are in accordance with the colors of the line spectra. The direction and tick scale of *x-z* axis for the nearfield profiles are uniformly shown by one profile in the green box for the sake of the concision, and the white lines depict the nanostructure in conformity to Figure S1. The colors of spectra indicate the *n*_super_ = 1 (blue), 1.46 (green), and 1.71 (red), while the black line spectra are the reference spectra of the Fe film with *n*_super_ = 1. The black vertical dashed line at *λ* = 581.6 nm denotes the condition of in-plane diffraction for all three different *n*_super_ (=1, 1.46, and 1.71), the blue vertical dashed line at *λ* = 398.4 nm denotes the in-plane diffraction for *n*_super_ = 1.46 for the blue line spectra, and the red vertical dashed lines at *λ* = 393.3 and 681.2 nm denote the in-plane diffraction for *n*_super_ = 1.71 for the red line spectra. The wavelengths of dips in the experimental transmission spectra (a, lower panel) are marked by square (at shorter wavelength) and circular marker (at longer wavelength) with their colors in accordance with the line colors of spectra. The corresponding wavelengths are indicated nearby the markers. To keep the uniformity, the same wavelength positions are also indicated by the same markers in the calculated transmission spectra (a, upper panel), FR spectra (b), and absorption spectra (c).

Figure 4 compares the calculated (upper panel) and experimental (lower panel) wavelength dependence of transmission (a) and FR (b) at *θ*_0_ = 0° for the Fe film on the Al NP array with *a* = 460 nm. The transmission spectra with *a* = 460 nm in Figure 4a are very similar to the ones with *a* = 400 nm in Figure 3a, except for the different diffraction wavelengths and redshifted transmission dips because of increasing lattice constant *a*. Similarly, we obtained the peaks and dips in the experimental FR spectra due to the lattice mode and LSPRs: peak 0.24° at *λ* = 450 nm, peak 0.36° at *λ* = 575 nm, dip 0.34° at *λ* = 595 nm, and peak 0.60° at *λ* = 681 nm for *n*_super_ = 1; peak 0.08° at *λ* = 435 nm, peak 0.16° at *λ* = 580 nm, dip −0.09° at *λ* = 604 nm, peak 0.69° at *λ* = 735 nm for *n*_super_ = 1.46; peak 0.08° at *λ* = 433 nm, peak 0.11° at *λ* = 580 nm, dip 0.06° at *λ* = 604 nm, peak 0.25° at *λ* = 687 nm, dip 0.21° at *λ* = 708 nm, peak 0.63° at *λ* = 800 nm for *n*_super_ = 1.71. The comparison between Figures 3b and 4b clearly shows that the Al NP array is better able to enhance FR with smaller lattice constant in the region 400–500 nm. As for longer wavelengths, notable enhancement in *θ*_F_ comparing to Fe film is still observed although the enhancement is smaller than the array with *a* = 400 nm. The reduced enhancement is linked to the decreased localized energy in the array with a larger lattice constant *a* by decreased NP density, although less influence is brought to the transmission spectra [40]. With respect to the calculated absorption spectra and plotted nearfield distribution at selected wavelengths in Figure 4c, a similar observation can be acquired as in Figure 3. And the enhanced MO phenomenon in Figure 4b can be well traced back to the resonant excitation of plasmonic modes shown by the markers for LSPRs and vertical dashed lines for SLRs in Figure 4c as explained above.

To evaluate the values of *θ*_F_ from the point of view of applications, we calculated the figure of merit (FOM) at certain wavelengths, defined as *|θ*_F_ *|T* ^0.5^, where *T* stands for the transmission value. The higher the FOM, the better it is in terms of the applicability to MO devices. Fe films on the arrays tend to show a higher FOM in the shorter wavelengths. For instance, the Fe film on the Al NP array shows a local maximum of FOM = 0.11° at *λ* = 410 nm, while that of a thin Fe film is very small (<0.01°). From the FOMs, it is manifest that the existence of Al NP arrays is beneficial for the enhancement of *θ*_F_ by the thin Fe film. In Table 1, we summarize the experimental FOMs of various plasmonic-magnetic systems in preceding works along with those in our study. In a random dispersion of Au NPs on Bi:YIG thin films, the enhancement was spectrally broad reflecting the width of the LSPPs on each Au NP (FOM is around 0.08°) [9]. In the periodic array of Au NPs, FOM becomes larger reaching a value of 0.11° at *λ* = 780 nm [16]. On the other hand, our results with Al NP arrays manifest the enhancement at the shortest wavelengths among the materials listed in the table because of the larger carrier density of Al and designed hybridized lattice mode around *λ* = 500 nm. The FOM is 0.11° at *λ* = 410 nm which is comparable to other experimental values for periodic plasmonic structures. By tuning the background, even higher FOM values are obtained at longer wavelength: 0.14° at *λ* = 580 nm for *n*_super_ = 1.46, and 0.39° at *λ* = 638 nm for *n*_super_ = 1.71. The exceptions to the previous statement are Au nanowire arrays on the Bi:YIG thin film [19] and Au nanowire arrays embedded in EuS thin film [20], where the working wavelength is beyond 700 nm. In these cases, the thin films act as a slab waveguide through which light–matter interaction was accelerated by additional round-trips propagation inside the MO materials.

**Table 1:** Preceding and present experimental works on enhanced Faraday rotation (FR) by film-based magnetic systems coupled to localized surface plasmon resonances (LSPRs).

Plasmonic structure		MO materials	*θ*_F_ (°)/*T*	FOM (°)/wavelength (nm)	Magnetic field (kOe)	Ref
Random Au NPs		Bi:YIG	0.18/0.17	0.07/644	–	[15]
Random Au NPs		Bi:YIG	−0.5/0.03	0.08/∼600	–	[9]
Au NP array		Bi:YIG	−0.3/0.15	0.11/780	2.0	[16]
Au nanowire array		Bi:YIG	0.80/0.36	0.48/963	1.4	[19]
Au NP array		Bi:YIG	0.5/0.07	0.13/700	–	[10]
1D Au grating		EuS	14/0.17	5.7/737	50	[20]
Al NP array (*a* = 400 nm)	*n*_super_ = 1	Fe	0.57/0.037	0.11/410	15	This study
	*n*_super_ = 1.46		0.8/0.029	0.14/580		
	*n*_super_ = 1.71		1.66/0.055	0.39/638		

–: Not indicated in the article.

## 4 Conclusions

Enhanced FR is potentially important on the development of miniatured optical devices such as isolators, circulators, sensors, and so forth. We designed a diffractive hexagonal array composed of Al NPs and enhanced the FR effect of a thin Fe film deposited on the top of the array. The tunable FR effects in the whole visible range from 400 to 800 nm were demonstrated by changing the lattice constant and the dielectric environment. The maxima appeared in the FR spectra are associated with the hybridized lattice modes. The varying dielectric environment around the Al NP can further tune the intensities and spectral regions of enhanced FR from blue to red region, which is ascribed to the shifting of LSPR of single Al NP in different backgrounds. By increasing the lattice constant, a weaker enhancement was observed due to the reduced energy confined in the sparse NP array. Our study clarified the advantages of Al NP array to drive the boosted MO effect from the widely studied green region into the UV-blue range that cannot be achieved by Au nanostructures reported thus far. Lastly, the hexagonal/honeycomb lattice is a complicated nanostructure, and different nature (collinear or non-collinear) of the diffraction modes can be excited depending on the mutual orientation between the lattice and the polarization of the incoming light [36, 37]. Here, we mainly pay our attention on FR at normal incidence, but further investigation on this feature will be physically important on unveiling the enhancement mechanism in other MO systems for polar and longitudinal MO Kerr effect.

## References

[1] Zvezdin A. K., Kotov V. A. (1997). *Modern Magnetooptics and Magnetooptical Materials*.

[2] Armelles G., Cebollada A., García-Martín A., González M. U. (2013). Magnetoplasmonics: combining magnetic and plasmonic functionalities. *Adv. Opt. Mater.*.

[3] Floess D., Giessen H. (2018). Nonreciprocal hybrid magnetoplasmonics. *Rep. Prog. Phys.*.

[4] Maccaferri N., Zubritskaya I., Razdolski I. (2020). Nanoscale magnetophotonics. *J. Appl. Phys.*.

[5] Krinchik G. S. (1964). Ferromagnetic Hall effect at optical frequencies and inner effective magnetic field of ferromagnetic metals. *J. Appl. Phys*.

[6] Maccaferri N., Gregorczyk K. E., de Oliveira T. V. A. G. (2015). Ultrasensitive and label-free molecular-level detection enabled by light phase control in magnetoplasmonic nanoantennas. *Nat. Commun.*.

[7] Katayama T., Suzuki Y., Awano H., Nishihara Y., Koshizuka N. (1988). Enhancement of the magneto-optical Kerr rotation in Fe/Cu bilayered films. *Phys. Rev. Lett.*.

[8] Hermann C., Kosobukin V. A., Lampel G., Peretti J., Safarov V. I., Bertrand P. (2001). Surface-enhanced magneto-optics in metallic multilayer films. *Phys. Rev. B*.

[9] Uchida H., Mizutani Y., Nakai Y., Fedyanin A. A., Inoue M. (2011). Garnet composite films with Au particles fabricated by repetitive formation for enhancement of Faraday effect. *J. Phys. D Appl. Phys.*.

[10] Belotelov V. I., Doskolovich L. L., Zvezdin A. K. (2007). Extraordinary magneto-optical effects and transmission through metal-dielectric plasmonic systems. *Phys. Rev. Lett.*.

[11] Tkachuk S., Lang G., Krafft C., Rabin O., Mayergoyz I. (2011). Plasmon resonance enhancement of Faraday rotation in thin garnet films. *J. Appl. Phys.*.

[12] Tomita S., Kato T., Tsunashima S., Iwata S., Fujii M., Hayashi S. (2006). Magneto-optical Kerr effects of yttrium-iron garnet thin films incorporating gold nanoparticles. *Phys. Rev. Lett.*.

[13] Jain P. K., Xiao Y., Walsworth R., Cohen A. E. (2009). Surface plasmon resonance enhanced magneto-optics (supremo): Faraday rotation enhancement in gold-coated iron oxide nanocrystals. *Nano Lett.*.

[14] Wang L., Clavero C., Huba Z. (2011). Plasmonics and enhanced magneto-optics in core−shell Co−Ag nanoparticles. *Nano Lett.*.

[15] Uchida H., Masuda Y., Fujikawa R., Baryshev A. V., Inoue M. (2009). Large enhancement of Faraday rotation by localized surface plasmon resonance in Au nanoparticles embedded in Bi: YIG film. *J. Magn. Magn Mater.*.

[16] Baryshev A. V., Merzlikin A. M. (2016). Tunable plasmonic thin magneto-optical wave plate. *J. Opt. Soc. Am. B*.

[17] Murai S., Yao S., Nakamura T. (2012). Modified Faraday rotation in a three-dimensional magnetophotonic opal crystal consisting of maghemite/silica composite spheres. *Appl. Phys. Lett.*.

[18] Caicedo J. M., Pascu O., López-García M. (2011). Magnetophotonic response of three-dimensional opals. *ACS Nano*.

[19] Chin J. Y., Steinle T., Wehlus T. (2013). Nonreciprocal plasmonics enables giant enhancement of thin-film Faraday rotation. *Nat. Commun.*.

[20] Belotelov V. I., Kreilkamp L. E., Kalish A. N. (2014). Magnetophotonic intensity effects in hybrid metal-dielectric structures. *Phys. Rev. B*.

[21] Floess D., Chin J. Y., Kawatani A. (2015). Tunable and switchable polarization rotation with non-reciprocal plasmonic thin films at designated wavelengths. *Light Sci. Appl.*.

[22] Floess D., Hentschel M., Weiss T. (2017). Plasmonic analog of electromagnetically induced absorption leads to giant thin film Faraday rotation of 14^o^. *Phys. Rev. X*.

[23] Murai S., Verschuuren M. A., Lozano G., Pirruccio G., Rodriguez S. R. K., Rivas J. G. (2013). Hybrid plasmonic-photonic modes in diffractive arrays of nanoparticles coupled to light-emitting optical waveguides. *Opt. Express*.

[24] Kravets V. G., Kabashin A. V., Barnes W. L., Grigorenko A. N. (2018). Plasmonic surface lattice resonances: a review of properties and applications. *Chem. Rev.*.

[25] Auguié B., Bendana X. M., Barnes W. L., Garcia de Abajo F. J. (2010). Diffractive arrays of gold nanoparticles near an interface: critical role of the substrate. *Phys. Rev. B*.

[26] Belotelov V. I., Akimov I. A., Pohl M. (2011). Enhanced magneto-optical effects in magnetoplasmonic crystals. *Nat. Nanotechnol.*.

[27] Sadeghi S., Hamidi S. M. (2018). Enhanced Faraday rotation in one dimensional magneto-plasmonic structure due to Fano resonance. *J. Magn. Magn Mater.*.

[28] Li D., Chen L., Lei C. (2016). Plasmon-enhanced magneto-optical activity in a nanostructure with circle annular arrays. *J. Opt. Soc. Am. B*.

[29] Luong H. M., Pham M. T., Nguyen T. D., Zhao Y. (2019). Enhanced resonant Faraday rotation in multilayer magnetoplasmonic nanohole arrays and their sensing application. *J. Phys. Chem. C*.

[30] Luong H. M., Pham M. T., Ai B., Nguyen T. D., Zhao Y. (2019). Magnetoplasmonic properties of Ag-Co composite nanohole arrays. *Phys. Rev. B*.

[31] Freire-Fernández F., Mansell R., van Dijken S. (2020). Magnetoplasmonic properties of perpendicularly magnetized [Co/Pt]_N_ nanodots. *Phys. Rev. B*.

[32] Maccaferri N., Bergamini L., Pancaldi M. (2016). Anisotropic nanoantenna-based magnetoplasmonic crystals for highly enhanced and tunable magneto-optical activity. *Nano Lett.*.

[33] Kataja M., Hakala T. K., Julku A., Huttunen M. J., van Dijken S., Törmä P. (2015). Surface lattice resonances and magneto-optical response in magnetic nanoparticle arrays. *Nat. Commun.*.

[34] Chen L., Gao J., Xia W. (2016). Tunable Fano resonance and magneto-optical response in magnetoplasmonic structure fabricated by pure ferromagnetic metals. *Phys. Rev. B*.

[35] Kolmychek I. A., Mamonov E. A., Gusev N. S., Sapozhnikov M. V., Golubev V. G., Murzina T. V. (2021). Resonant optical effects in composite Co/opal-based magnetoplasmonic structures. *Opt. Lett.*.

[36] Maccaferri N., Inchausti X., García-Martín A. (2015). Resonant enhancement of magneto-optical activity induced by surface plasmon polariton modes coupling in 2D magnetoplasmonic crystals. *ACS Photonics*.

[37] Rollinger M., Thielen P., Melander E. (2016). Light localization and magneto-optic enhancement in Ni antidot arrays. *Nano Lett.*.

[38] Martin J., Kociak M., Mahfoud Z., Proust J., Gérard D., Plain J. (2014). High-resolution imaging and spectroscopy of multipolar plasmonic resonances in aluminum nanoantennas. *Nano Lett.*.

[39] Zhang F., Martin J., Plain J. (2019). Long-term stability of plasmonic resonances sustained by evaporated aluminum nanostructures. *Opt. Mater. Express*.

[40] Zhang F., Tang F., Xu X., Adam P. M., Martin J., Plain J. (2020). Influence of order-to-disorder transitions on the optical properties of the aluminum plasmonic metasurface. *Nanoscale*.

[41] Zhang F., Plain J., Gerard D., Martin J. (2021). Surface roughness and substrate induced symmetry-breaking: influence on the plasmonic properties of aluminum nanostructure arrays. *Nanoscale*.

[42] Zhang F., Martin J., Murai S., Adam P. M., Plain J., Tanaka K. (2021). Evidence of the retardation effect on the plasmonic resonances of aluminum nanodisks in the symmetric/asymmetric environment. *Opt. Express*.

[43] de Sousa N., Froufe-Pérez L. S., Sáenz J. J., García-Martín A. (2016). Magneto-optical activity in high index dielectric nanoantennas. *Sci. Rep.*.

[44] Barsukova M. G., Shorokhov A. S., Musorin A. I., Neshev D. N., Kivshar Y. S., Fedyanin A. A. (2017). Magneto-optical response enhanced by Mie resonances in nanoantennas. *ACS Photonics*.

[45] Barsukova M. G., Musorin A. I., Shorokhov A. S., Fedyanin A. A. (2019). Enhanced magneto-optical effects in hybrid Ni-Si metasurfaces. *APL Photonics*.

[46] Kaihara T., Mizuguchi M., Takanashi K., Shimizu H. (2013). Magneto-optical properties and size effect of ferromagnetic metal nanoparticles. *Jpn. J. Appl. Phys*.

[47] Abendroth J. M., Solomon M. L., Barton D. R., El Hadri M. S., Fullerton E. E., Dionne J. A. (2020). Helicity-preserving metasurfaces for magneto-optical enhancement in ferromagnetic [Pt/Co]_N_ films. *Adv. Opt. Mater.*.

[48] Joannopoulos J. D., Meade R. D., Winn J. N. (1995). *Photonic Crystals: Molding the Flow of Light*.

